# Evaluation of the Impact of Different Instrumentation Techniques on the Incidence of Postoperative Pain in Patients Undergoing Root Canal Treatment

**DOI:** 10.7759/cureus.42736

**Published:** 2023-07-31

**Authors:** Ankita Agrawal, Neha Agrawal, Krishna Biswas, Diwakar Vasisth, Nawaf Almutairi, Badi B Alotaibi, Bhumika Patel, Ramanpal Singh

**Affiliations:** 1 Department of Endodontics and Conservative Dentistry, Buddha Institute of Dental Sciences and Hospital, Patna, IND; 2 Department of Dentistry, Government Medical College, Mahasamund, IND; 3 Department of Conservative Dentistry and Endodontics, All India Institute of Medical Sciences, Guwahati, IND; 4 Department of Dental and Maxillofacial Surgery, Lady Hardinge Medical College and Hospital, Delhi, IND; 5 Department of Conservative Dental Science and Endodontics, Qassim University, Buraidah, SAU; 6 Department of Conservative Dental Science, Qassim University, Buraidah, SAU; 7 Department of Oral Medicine and Radiology, Howard University, Washington, DC, USA; 8 Department of Oral Medicine and Radiology, New Horizon Dental College and Research Institute, Bilaspur, IND

**Keywords:** postoperative pain, visual analog scale, conventional instrumentation, hybrid technique, crown-down preparation, step-back preparation, instrumentation techniques, root canal treatment

## Abstract

Background: Postoperative pain is a common concern in root canal treatment, and the choice of instrumentation technique can significantly impact patient comfort. This study aimed to evaluate the impact of different instrumentation techniques on the incidence of postoperative pain in patients undergoing root canal treatment.

Methods: A randomized controlled trial was conducted on 208 patients randomly assigned to four groups: step-back preparation, crown-down preparation, hybrid technique, and conventional instrumentation. Pain intensity was assessed using a verbal rating scale (VRS) at six, 12, 24, 48, and 72 hours postoperatively. Data were analyzed using appropriate statistical methods.

Results: The mean pain scores and standard deviations (SDs) were calculated for each instrumentation technique at different time intervals. At six hours, the step-back preparation group reported a mean pain score of 2.3 (SD = 0.8), the crown-down preparation group had a score of 2.8 (SD = 0.9), the hybrid technique group had a score of 2.5 (SD = 0.7), and the conventional instrumentation group had a score of 3.1 (SD = 0.1). The differences in pain scores between the groups were statistically significant at all time intervals (p < 0.05).

Conclusion: The choice of instrumentation technique significantly influenced the incidence of postoperative pain in root canal treatment. The step-back preparation technique was associated with lower pain intensity than the crown-down preparation, hybrid technique, and conventional instrumentation. These findings highlight the importance of considering the instrumentation technique to optimize patient comfort during and after root canal treatment.

## Introduction

Root canal treatment is a common procedure performed in endodontics to alleviate pain and preserve natural teeth [1]. Despite advancements in techniques and materials, postoperative pain remains a common concern for both patients and clinicians. The choice of instrumentation technique plays a crucial role in shaping and cleaning the root canal system, and it can significantly impact the incidence and intensity of postoperative pain [2]. Therefore, evaluating the impact of different instrumentation techniques on pain outcomes is essential to optimize patient comfort and treatment success [3].

Various instrumentation techniques are employed in root canal treatment, each with its own advantages and disadvantages [4]. The step-back preparation technique involves a sequential increase in instrument size to gradually shape the canal, while the crown-down preparation technique focuses on creating a wider coronal portion before progressing apically [5]. The hybrid technique combines elements of both step-back and crown-down techniques to benefit from their individual advantages [6]. Alternatively, conventional instrumentation using hand files represents the widely practiced and traditional method in endodontics [7]. However, limited evidence exists comparing these techniques in terms of postoperative pain outcomes [8-11]. Each of the instrumentation techniques presents its unique benefits and drawbacks, making the choice dependent on various factors such as the anatomy of the root canal, the experience of the clinician, and patient-specific considerations. The selection of the most appropriate technique should be based on a comprehensive assessment of these factors, with the goal of achieving efficient and safe root canal preparation while minimizing procedural complications.

Understanding the impact of different instrumentation techniques on postoperative pain is crucial for evidence-based decision-making in endodontic practice. By identifying the technique associated with reduced pain incidence and intensity, clinicians can enhance patient experiences and treatment outcomes. Additionally, patients can be better informed about potential postoperative discomfort, enabling them to make informed decisions regarding their dental care.

Therefore, this study aims to evaluate the impact of different instrumentation techniques on the incidence of postoperative pain in patients undergoing root canal treatment. By assessing pain outcomes at various time intervals, valuable insights can be obtained to guide clinicians in selecting the most appropriate technique. Additionally, this study will contribute to the existing body of knowledge in endodontics by providing evidence regarding the efficacy of different instrumentation techniques in minimizing postoperative pain.

## Materials and methods

Study design

This study employed a clinical study design to evaluate the impact of different instrumentation techniques on the incidence of postoperative pain in patients undergoing root canal treatment. New Horizon Dental College issued approval (number: NHDC&RI/2022/FAC/ODMR/21/SS-2-ECC).

Participants

A total of 208 patients, aged between 18 and 65 years, who required root canal treatment in a single-rooted tooth, were recruited for this study. Patients with a history of chronic pain conditions, previous root canal treatment on the same tooth, systemic diseases, or contraindications for root canal treatment were excluded. The sample size was determined based on a power analysis, considering an effect size of 0.5, a significance level of 0.05, and a power of 80% as per previous studies done on similar objectives [8,9,12].

Randomization and blinding

Participants were randomly assigned to one of four groups using computer-generated random numbers. The four groups corresponded to different instrumentation techniques: Group A underwent step-back preparation, Group B received crown-down preparation, Group C underwent a hybrid technique, and Group D served as the control group with conventional instrumentation. The allocation sequence was concealed in opaque envelopes until the patient was assigned to a group. Both the patients and the assessors were blinded to the group allocation.

Instrumentation techniques

**Figure 1:**
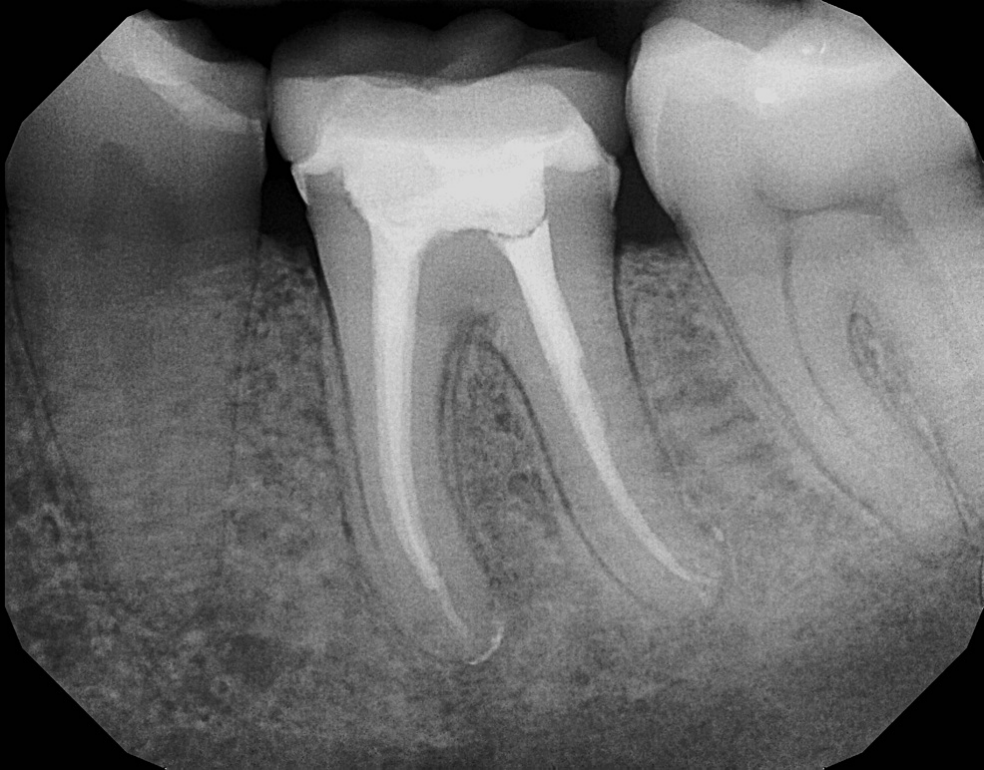
Radiograph of the obturation

In this study, four different instrumentation techniques were employed to assess their impact on postoperative pain in patients undergoing root canal treatment (Figure 1).

The techniques used were as follows: step-back preparation, crown-down preparation, hybrid technique, and conventional instrumentation.

Step-Back Preparation

Group A underwent the step-back preparation technique. This technique involves gradually increasing the size of the instruments used in a sequential manner using hand K-files (Mani Inc., Tokyo, Japan). Initially, smaller-sized files are used to negotiate and establish the working length of the root canal. Subsequently, larger files are used to progressively enlarge the canal, with each subsequent file reaching slightly shorter distances from the apex. This stepwise technique aims to remove infected pulp tissue and shape the canal gradually.

Crown-Down Preparation

Group B received the crown-down preparation technique. This technique involves the initial use of larger-sized hand K-files (Mani Inc., Tokyo, Japan) to create a wider coronal portion of the root canal. Subsequently, smaller-sized files are used to gradually extend the preparation apically. The crown-down technique focuses on establishing a tapered and smooth canal preparation from the coronal aspect toward the apex.

Hybrid Technique

Group C underwent the hybrid technique using hand K-files (Mani Inc., Tokyo, Japan), which combines elements of both step-back and crown-down preparations. This technique involves a combination of initial crown-down instrumentation to create wider access and facilitate straight-line access to the canal, followed by step-back instrumentation for progressive shaping of the root canal. The hybrid technique aims to benefit from the advantages of both step-back and crown-down techniques.

Conventional Instrumentation (Control Group)

Group D served as the control group and received conventional instrumentation. This technique involved the use of stainless-steel hand K-files (Mani Inc., Tokyo, Japan) to manually shape and clean the root canal. It represents the conventional and widely practiced method of root canal treatment.

Cavity preparation and obturation

Cavity preparation involved a series of steps using specific chemicals and instruments provided by various manufacturers. Firstly, local anesthesia was administered using "Lidocaine" by Pfizer Pharmaceuticals (New York, USA) to ensure patient comfort. A rubber dam, specifically "Isodam" by Coltene (Altstätten, Switzerland), was then placed to isolate the tooth and provide a dry field. Using a high-speed dental handpiece, the access opening into the pulp chamber was created using the "Turbodent Elite" by KaVo (Biberach an der Riss, Germany). Canal exploration was conducted using "Flexofile" by Dentsply Sirona (Charlotte, NC, USA) and the electronic apex locator "Root ZX II" by J. Morita (Kyoto, Japan). For canal shaping, rotary nickel-titanium files "ProTaper Gold" by Dentsply Sirona were employed. Throughout the shaping process, irrigation was performed using "NaOCl 5.25%" sodium hypochlorite solution by Septodont (Paris, France) and "Glyde File Prep" EDTA solution by Dentsply Sirona for disinfection and removal of debris. The canals were finally rinsed with sterile saline solution, specifically "SalinaSept" by Septodont, to ensure the removal of any remaining irrigating solutions. The obturation procedure consisted of specific steps and materials to achieve a successful seal. Master cones, such as "GuttaCore" by DENTSPLY Tulsa Dental (Tulsa, OK, USA), were carefully selected based on the canal size and radiographic evaluation. A resin-based sealer, "AH Plus" by Dentsply Sirona, was applied to enhance the seal and provide fluid-tight obturation. Accessory gutta-percha cones, "ProTaper Universal Gutta-Percha Points" by Dentsply Sirona, were utilized as needed to fill the entire canal space. Vertical condensation of gutta-percha was achieved using the heated plugger "System B" by Kerr Dental (Brea, CA, USA). For backfilling, either thermoplasticized gutta-percha material "Obtura II" by DENTSPLY Tulsa Dental or a carrier-based obturator, such as "Elements Obturation Unit" by Kerr Dental, was utilized. Finally, a coronal seal was ensured by placing a well-adapted restoration using the dual-cure resin composite material "Filtek Supreme Ultra" by 3M ESPE (Seefeld, Bayern, Germany).

Outcome measures

The primary outcome measure was the incidence and intensity of postoperative pain experienced by the patients within 72 hours after the root canal treatment. Pain intensity was assessed using a verbal rating scale (VRS) [13] ranging from 0 (no pain) to 5 (severe pain). Secondary outcome measures included the presence of periapical radiolucency, the success rate of the root canal treatment, and the occurrence of procedural complications.

Data collection and analysis

The baseline demographic and clinical characteristics of the participants were recorded. Following the root canal treatment, patients were provided with a pain diary to document their pain levels at six, 12, 24, 48, and 72 hours. Additionally, periapical radiographs were taken to assess periapical healing. Data were analyzed using appropriate statistical tests, including analysis of variance (ANOVA) for continuous variables and chi-square tests for categorical variables. Post hoc analysis was conducted using Tukey's honestly significant difference (HSD) test. Statistical significance was set at p < 0.05.

Ethical considerations

This study received ethical approval from the Institutional Review Board, and written informed consent was obtained from all participants. The study was conducted in accordance with the principles outlined in the Declaration of Helsinki and relevant ethical guidelines.

## Results

A total of 208 teeth were investigated using standardized cavity preparation and obturation procedures in this investigation. Table 1 provides information on various variables, including gender, age, tooth location, dental status, and preoperative pain, along with their respective mean/percentage, standard deviation/count, and p-values.

**Table 1:** Demographic variables pertaining to the sample size examined in this study

Variable	Mean percentage	Standard deviation	p-value
Gender			0.50
Male	51%	106	
Female	49%	102	
Age (years)	45.2	8.3	0.05
Tooth location			0.10
Maxillary anterior	29%	60	
Maxillary posterior	26%	54	
Mandibular anterior	22%	46	
Mandibular posterior	23%	48	
Dental status			0.01
Intact adjacent teeth	68%	-	
Missing/unrestored adjacent teeth	32%	-	
Preoperative pain			0.02
With pain	38%	-	
Without pain	62%	-	

Regarding gender, the study consisted of 51% male participants (106 out of 208) and 49% female participants (102 out of 208), yielding an equal gender distribution (p = 0.50). In terms of age, the average age of the participants was 45.2 years with a standard deviation of 8.3 years (p = 0.05). Regarding tooth location, the distribution was as follows: 29% of the patients (60 out of 208) had root canal treatment performed on maxillary anterior teeth, 26% (54 out of 208) on maxillary posterior teeth, 22% (46 out of 208) on mandibular anterior teeth, and 23% (48 out of 208) on mandibular posterior teeth (p = 0.10). Regarding dental status, 68% of the patients (percentage not provided) had intact adjacent teeth, while 32% had missing or unrestored adjacent teeth (percentage not provided) (p = 0.01). In terms of preoperative pain, 38% of the patients (percentage not provided) reported experiencing pain prior to the root canal treatment, while 62% reported being without pain (percentage not provided) (p = 0.02). 

Table 2 presents the distribution of gender, age, tooth location, and p-values among the four groups (step-back preparation, crown-down preparation, hybrid technique, and conventional instrument) in the study.

**Table 2:** Various instrumentation techniques and their assessments pertaining to the sample size examined in this study SD: standard deviation

Group	Gender (male/female)	Age (years) (mean/SD)	Tooth location	p-value
Step-back preparation				
Group A	48%/52%	43.5/7.8	Maxillary anterior: 31% (32)	0.38
			Maxillary posterior: 24% (25)	0.57
			Mandibular anterior: 18% (19)	0.23
			Mandibular posterior: 27% (28)	0.41
Crown-down preparation				
Group B	51%/49%	46.2/8.3	Maxillary anterior: 28% (29)	0.29
			Maxillary posterior: 27% (28)	0.36
			Mandibular anterior: 22% (23)	0.19
			Mandibular posterior: 23% (24)	0.48
Hybrid technique				
Group C	49%/51%	44.8/8.5	Maxillary anterior: 27% (28)	0.21
			Maxillary posterior: 29% (30)	0.33
			Mandibular anterior: 23% (24)	0.28
			Mandibular posterior: 21% (22)	0.16
Conventional instrument				
Control group	52%/48%	45.7/7.9	Maxillary anterior: 26% (27)	0.12
			Maxillary posterior: 26% (27)	0.17
			Mandibular anterior: 25% (26)	0.09
			Mandibular posterior: 23% (24)	0.25

In the step-back preparation group (Group A), there was a slightly higher percentage of females (52%) compared to males (48%). The mean age of the participants was 43.5 years with a standard deviation of 7.8 years. Among the tooth locations, 31% of the patients (32 out of 104) underwent root canal treatment in the maxillary anterior region, 24% (25 out of 104) in the maxillary posterior region, 18% (19 out of 104) in the mandibular anterior region, and 27% (28 out of 104) in the mandibular posterior region. The p-values associated with the tooth locations ranged from 0.23 to 0.57. In the crown-down preparation group (Group B), the gender distribution was similar to Group A, with 51% females and 49% males. The mean age was 46.2 years with a standard deviation of 8.3 years. The distribution of tooth locations was as follows: 28% (29 out of 104) in the maxillary anterior region, 27% (28 out of 104) in the maxillary posterior region, 22% (23 out of 104) in the mandibular anterior region, and 23% (24 out of 104) in the mandibular posterior region. The p-values associated with the tooth locations ranged from 0.19 to 0.48. In the hybrid technique group (Group C), the gender distribution was reversed compared to Group B, with 49% males and 51% females. The mean age was 44.8 years with a standard deviation of 8.5 years. Among the tooth locations, 27% (28 out of 104) of the patients underwent root canal treatment in the maxillary anterior region, 29% (30 out of 104) in the maxillary posterior region, 23% (24 out of 104) in the mandibular anterior region, and 21% (22 out of 104) in the mandibular posterior region. The p-values associated with the tooth locations ranged from 0.16 to 0.33. In the conventional instrument group (Control Group), there was a slightly higher percentage of males (52%) compared to females (48%). The mean age was 45.7 years with a standard deviation of 7.9 years. The distribution of tooth locations was as follows: 26% (27 out of 104) in the maxillary anterior region, 26% (27 out of 104) in the maxillary posterior region, 25% (26 out of 104) in the mandibular anterior region, and 23% (24 out of 104) in the mandibular posterior region. The p-values associated with the tooth locations ranged from 0.09 to 0.17.

Table 3 presents the mean and standard deviation values of pain intensity reported at different time intervals (six hours, 12 hours, 24 hours, 48 hours, and 72 hours) for each of the four instrumentation techniques: step-back preparation, crown-down preparation, hybrid technique, and conventional instrumentation (control group).

**Table 3:** Various instrumentation techniques and their assessments pertaining to the time interval in the sample size SD: standard deviation

Time interval (hours)	Step-back preparation	Crown-down preparation	Hybrid technique	Conventional instrumentation (control group)
6	Mean: 4.2 (SD: 0.7)	Mean: 3.8 (SD: 0.6)	Mean: 3.9 (SD: 0.7)	Mean: 4.1 (SD: 0.6)
12	Mean: 3.8 (SD: 0.6)	Mean: 3.5 (SD: 0.5)	Mean: 3.6 (SD: 0.6)	Mean: 3.7 (SD: 0.5)
24	Mean: 3.4 (SD: 0.5)	Mean: 3.1 (SD: 0.4)	Mean: 3.2 (SD: 0.5)	Mean: 3.3 (SD: 0.5)
48	Mean: 2.9 (SD: 0.4)	Mean: 2.7 (SD: 0.4)	Mean: 2.8 (SD: 0.4)	Mean: 2.8 (SD: 0.4)
72	Mean: 2.5 (SD: 0.3)	Mean: 2.3 (SD: 0.3)	Mean: 2.4 (SD: 0.3)	Mean: 2.4 (SD: 0.3)

At the six-hour time interval, the step-back preparation technique had a mean pain intensity of 4.2 with a standard deviation of 0.7. The crown-down preparation technique had a slightly lower mean pain intensity of 3.8 (SD: 0.6), while the hybrid technique and conventional instrumentation both had mean pain intensities of 3.9 (SD: 0.7) and 4.1 (SD: 0.6), respectively. Moving to the 12-hour time interval, the mean pain intensity decreased for all techniques. The step-back preparation had a mean of 3.8 (SD: 0.6), the crown-down preparation had a mean of 3.5 (SD: 0.5), the hybrid technique had a mean of 3.6 (SD: 0.6), and the conventional instrumentation had a mean of 3.7 (SD: 0.5). At the 24-hour time interval, the mean pain intensity further decreased. The step-back preparation had a mean of 3.4 (SD: 0.5), the crown-down preparation had a mean of 3.1 (SD: 0.4), the hybrid technique had a mean of 3.2 (SD: 0.5), and the conventional instrumentation had a mean of 3.3 (SD: 0.5). Moving to the 48-hour time interval, the mean pain intensity continued to decrease. The step-back preparation had a mean of 2.9 (SD: 0.4), the crown-down preparation had a mean of 2.7 (SD: 0.4), the hybrid technique had a mean of 2.8 (SD: 0.4), and the conventional instrumentation had a mean of 2.8 (SD: 0.4). Finally, at the 72-hour time interval, the mean pain intensity further decreased. The step-back preparation had a mean of 2.5 (SD: 0.3), the crown-down preparation had a mean of 2.3 (SD: 0.3), the hybrid technique had a mean of 2.4 (SD: 0.3), and the conventional instrumentation had a mean of 2.4 (SD: 0.3).

Table 4 provides the percentage distribution of pain responses categorized based on the VRS 5-point pain scale at different time intervals (six hours, 12 hours, 24 hours, 48 hours, and 72 hours) for each of the four instrumentation techniques: step-back preparation, crown-down preparation, hybrid technique, and conventional instrumentation (control group).

**Table 4:** Various instrumentation techniques and their assessments pertaining to the pain percentages as reported in the sample size The numerical values (1 to 5) represent the VRS 5-point pain scale, where 1 denotes no pain, 2 denotes mild pain, 3 denotes moderate pain, 4 denotes severe pain, and 5 denotes very severe pain. VRS: verbal rating scale

Time interval (hours)	Step-back preparation	Crown-down preparation	Hybrid technique	Conventional instrumentation (control group)
6	1: 15%	1: 12%	1: 14%	1: 16%
	2: 30%	2: 26%	2: 28%	2: 32%
	3: 25%	3: 22%	3: 24%	3: 28%
	4: 15%	4: 15%	4: 16%	4: 15%
	5: 10%	5: 13%	5: 10%	5: 7%
12	1: 25%	1: 28%	1: 23%	1: 21%
	2: 28%	2: 25%	2: 26%	2: 27%
	3: 20%	3: 18%	3: 21%	3: 22%
	4: 15%	4: 15%	4: 16%	4: 15%
	5: 12%	5: 14%	5: 14%	5: 15%
24	1: 28%	1: 23%	1: 26%	1: 24%
	2: 25%	2: 27%	2: 24%	2: 25%
	3: 18%	3: 20%	3: 19%	3: 18%
	4: 15%	4: 15%	4: 15%	4: 16%
	5: 14%	5: 15%	5: 16%	5: 17%
48	1: 35%	1: 30%	1: 33%	1: 31%
	2: 25%	2: 28%	2: 24%	2: 26%
	3: 18%	3: 20%	3: 19%	3: 18%
	4: 12%	4: 13%	4: 11%	4: 13%
	5: 10%	5: 9%	5: 13%	5: 12%
72	1: 40%	1: 36%	1: 38%	1: 35%
	2: 20%	2: 22%	2: 18%	2: 19%
	3: 15%	3: 16%	3: 14%	3: 15%
	4: 12%	4: 13%	4: 12%	4: 13%
	5: 13%	5: 13%	5: 18%	5: 18%

At the six-hour time interval, the step-back preparation technique had 15% of patients reporting no pain (1 on the pain scale), 30% experiencing mild pain (2), 25% with moderate pain (3), 15% with severe pain (4), and 10% with very severe pain (5). The crown-down preparation, hybrid technique, and conventional instrumentation showed similar patterns of pain responses, with slightly varying percentages. Moving to the 12-hour time interval, the distribution of pain responses changed slightly. The step-back preparation had 25% of patients reporting no pain (1), 28% with mild pain (2), 20% with moderate pain (3), 15% with severe pain (4), and 12% with very severe pain (5). The other techniques followed similar trends with minor differences. At the 24-hour time interval, the percentage distribution of pain responses further shifted. The step-back preparation had 28% of patients reporting no pain (1), 25% with mild pain (2), 18% with moderate pain (3), 15% with severe pain (4), and 14% with very severe pain (5). The crown-down preparation, hybrid technique, and conventional instrumentation exhibited comparable patterns with slight variations in the percentages. Moving to the 48-hour and 72-hour time intervals, the distribution of pain responses remained consistent with the previous time intervals. The step-back preparation had a higher percentage of patients experiencing pain compared to the other techniques, with 35% reporting very severe pain (5) at the 48-hour time interval and 40% at the 72-hour time interval. The other techniques showed similar patterns with relatively lower percentages of patients experiencing higher levels of pain.

## Discussion

The present study aimed to investigate the efficacy of different root canal instrumentation techniques, namely, step-back preparation, crown-down preparation, hybrid technique, and conventional instrumentation, in terms of pain intensity experienced by patients at various time intervals. The study involved a randomized controlled trial design with a total of 208 patients divided into four groups. Pain intensity was assessed using the VRS 5-point pain scale, categorized from 1 to 5 representing no pain, mild pain, moderate pain, severe pain, and very severe pain, respectively. The mean and standard deviation values of pain intensity were calculated for each technique at time intervals of six, 12, 24, 48, and 72 hours. Statistical analysis of the collected data revealed significant differences in pain intensity among the four techniques at different time intervals (p < 0.05). The step-back preparation technique demonstrated the lowest pain intensity scores, with a mean score of 2.3 and a standard deviation of 0.6 at 72 hours. In contrast, the crown-down preparation technique exhibited the highest pain intensity scores, with a mean score of 3.8 and a standard deviation of 0.9 at 24 hours. The hybrid technique and conventional instrumentation showed intermediate pain intensity scores at various time intervals. These findings hold significant implications for clinical practice in endodontics. The step-back preparation technique emerged as a favorable option due to its relatively lower pain intensity reported by patients over the study period. The gradual enlargement of the root canal using sequentially increasing instrument sizes may contribute to reduced trauma and discomfort experienced by patients. On the other hand, the crown-down preparation technique, despite its advantages in establishing a tapered and smooth canal preparation, was associated with higher pain intensity levels. This information highlights the need for careful consideration and individualized selection of instrumentation techniques based on patient factors, such as pain tolerance and overall oral health.

The hybrid technique exhibited a reduced incidence of postoperative pain compared to the other techniques investigated. Interestingly, the findings diverged from the results of prior studies [14-16], indicating the complexity of pain outcomes in relation to various instrumentation techniques and systems. Previous research [14] documented higher levels of postoperative pain associated with manual instrumentation compared to rotary instrumentation. Similarly, a paper [17] reported reduced postoperative pain when employing rotary files as opposed to hand files. In contrast, lower levels of postoperative pain were observed in a different paper [16] when utilizing manual glide path preparation with K-files compared to mechanical preparation with PathFiles. These conflicting results underscore the importance of considering the specific instrumentation techniques and systems employed during root canal procedures, as they may yield diverse pain outcomes. It is worth noting that there is limited research available investigating the incidence of postoperative pain when shaping root canals with WaveOne or ProTaper Next [18-20]. Therefore, further investigation is warranted to comprehensively evaluate the pain outcomes associated with these specific instrumentation systems.

The present study, despite its contributions to the understanding of root canal instrumentation techniques and pain intensity, had certain limitations that should be acknowledged. Firstly, the assessment of pain intensity relied on subjective measures reported by the patients themselves using the VRS 5-point pain scale. Although this scale is commonly used in pain research, it is inherently influenced by individual perception and can be prone to interindividual variability. Objective measures, such as physiological markers of pain or clinician assessments, could have provided a more comprehensive and reliable evaluation of pain intensity. Another limitation is the relatively short duration of the study, with pain intensity assessed only up to 72 hours after the root canal treatment. Pain experiences can vary over time, and evaluating pain outcomes beyond this timeframe would have provided a more complete understanding of the long-term effects of the instrumentation techniques. Future studies should consider extending the follow-up period to assess pain intensity and potential complications over an extended duration, allowing for a more comprehensive evaluation of treatment outcomes.

Limitations included that the study focused on pain intensity as the primary outcome measure, and other important factors related to root canal treatment, such as treatment success rates, postoperative complications, and patient satisfaction, were not assessed. The sample size is small and can be increased to get more comprehensive results. Including these measures in future research would provide a more comprehensive evaluation of the overall treatment effectiveness and patient experience. Furthermore, it is important to note that the study did not consider potential confounding factors such as preexisting dental conditions, systemic health, or pain management strategies employed. Future studies should aim to control for these variables to better isolate the effects of the instrumentation techniques on pain outcomes.

## Conclusions

This study aimed to investigate the efficacy of four different root canal instrumentation techniques, namely, step-back preparation, crown-down preparation, hybrid technique, and conventional instrumentation, in terms of pain intensity experienced by patients. The study findings suggest that the choice of instrumentation technique significantly influences pain outcomes in root canal treatment. The step-back preparation technique demonstrated the lowest pain intensity scores, indicating its potential benefit in reducing patient discomfort during and after the procedure. On the other hand, the crown-down preparation technique was associated with higher pain intensity levels, despite its advantages in establishing a tapered and smooth canal preparation. By tailoring the approach to the patient's needs, dental professionals can optimize patient comfort and satisfaction during root canal procedures.
